# Insights From a Mixed Methods Analysis of 3 Health Technologies Used in Patients With Parkinson Disease: Mixed Methods Study

**DOI:** 10.2196/67986

**Published:** 2025-08-01

**Authors:** Daniel Pérez-Rangel, Mariana H G Monje, Sylvie Grosjean, Martin Srp, Laura Antunes, Raquel Bouça-Machado, Ricardo Cacho, Sergio Domínguez Rodríguez, John Inocentes, Timothy Lynch, Argyri Tsakanika, Dimitrios Fotiadis, George Rigas, Evžen Růžička, Joaquim J Ferreira, Angelo Antonini, Norberto Malpica, Tiago Mestre, Álvaro Sánchez-Ferro, iCARE-PD Consortium

**Affiliations:** 1 Movement Disorders Unit, Neurology Department, Hospital Universitario 12 De Octubre, Av. de Córdoba, s/n, Usera, Madrid, 28041, Spain, 34 913908000; 2 Ken and Ruth Davee Department of Neurology, Northwestern University, Feinberg School of Medicine, Chicago, IL, United States; 3 Department of Communication, Com&Tech Innovations Lab, University of Ottawa, Ottawa, ON, Canada; 4 Department of Neurology and Centre of Clinical Neuroscience, First Faculty of Medicine, Charles University, Prague, Czech Republic; 5 CNS - Campus Neurológico Torres Vedras, Lisbon, Portugal; 6 LAIMBIO, Laboratorio de Análisis de Imagen Médica y Biometría, Universidad Rey Juan Carlos, Madrid, Spain; 7 Dublin Neurological Institute, Mater Misericordiae University Hospital, Dublin, Ireland; 8 PD Neurotechnology, Athens, Greece; 9 Parkinson and Movement Disorders Unit, Department of Neurosciences, Padova University, Padova, Italy; 10 Parkinson’s Disease and Movement Disorders Center, Division of Neurology, Department of Medicine, The Ottawa Hospital Research Institute, University of Ottawa's Brain and Mind Research Institute, Ottawa, ON, Canada

**Keywords:** technology-enabled care, usability, acceptability, Parkinson disease

## Abstract

**Background:**

The transition to a patient-centered integrated care model in Parkinson disease (PD) highlights the crucial role of technology. “Technology-enabled care” (TEC) supports diagnosis, disease tracking, self-care education, and care team communication. However, gaps remain in developing and evaluating patient-centered TEC solutions.

**Objective:**

This study aims to evaluate the usability and acceptability of 3 health technologies for PD and discuss the significance of the results.

**Methods:**

This multicenter international study was conducted from December 2020 to September 2023 across 5 tertiary PD centers. Participants included individuals diagnosed with PD who were recruited through these centers. Each participant provided informed consent before enrollment. The study assessed the usability and acceptability of 3 different health technologies designed to support PD management. The System Usability Scale (SUS) was used as the primary quantitative measure, with scores ranging from 0 to 100, with higher scores indicating greater usability. Additionally, participants completed a custom usability and acceptability survey, which included Likert-scale questions and open-ended qualitative feedback. To ensure a comprehensive evaluation, structured user testing sessions were conducted. Participants interacted with each technology under guided conditions, followed by independent use in their home environment for a specified period. Data were collected at baseline and after the trial period to assess any changes in user perception. Qualitative thematic analysis of free-text responses was performed to identify key themes related to user experience, perceived benefits, and challenges. Two independent researchers analyzed the qualitative data to ensure reliability and consistency in theme extraction.

**Results:**

The study included 43 people with PD, of whom 15 were female. The median age of participants was 67.0 (IQR 59.9‐71.5) years, and the median disease duration was 9.6 (IQR 5.0‐13.7) years. The 3 health technologies demonstrated acceptable usability, with median SUS scores ranging from 74.0 to 82.5. Participants expressed a generally positive attitude toward TEC, with a strong interest in continued use. Users particularly valued confidence in navigating the technology and its role in facilitating disease management. The qualitative analysis highlighted several challenges. Users frequently mentioned the need for improved technical support, clearer instructional materials, and simplified report formats to enhance interpretability. Some participants experienced difficulties with initial setup and required assistance, emphasizing the importance of user-friendly onboarding processes.

**Conclusions:**

Our study underscores the importance of incorporating patient perspectives in the development of health technologies for PD. Positive user experiences demonstrate the potential of TEC to enhance disease management, but addressing usability challenges remains critical. Future efforts should focus on refining user interfaces, providing comprehensive technical support, and ensuring clear, accessible instructions to maximize adoption and long-term engagement. By prioritizing these aspects, TEC can play a pivotal role in advancing patient-centered health care solutions for PD.

## Introduction

The management of Parkinson disease (PD) requires a comprehensive approach. At the personal level, the relentless clinical progression in PD implies progressively more complex care needs [1]. At the health care system level, the increase in PD prevalence in the population warrants the development of care delivery strategies. The shift of the care of people with PD from a reactive, center-based care [2] to a patient-centered integrated approach is a novel strategy that may address these challenges. This strategy enhances continuity of care and integration with the patient’s home environment [3], thereby reducing the burden of care and improving care access [4].

Technology, such as telemedicine and self-care apps, can play an instrumental role in implementing a patient-centered care model [5,6]. Initiatives like the iCARE-PD consortium [1] aim to develop user-friendly toolkits for constructing local integrated care networks focused on home-based, community-centered care, enabling self-management support for patients with PD [1], in which technology-enabled care (TEC) is fundamental. Nevertheless, the development and implementation of TEC has its own challenges, including usability and acceptability of the use of technology by people with PD and care partners.

To address these challenges, patient-centric approaches are favored as they require the end user to be actively involved in the development of health technologies [6]. This approach enhances the translational potential of digital health solutions and improves long-term patient adherence [7].

The technologies were chosen based on their ability to support “Technology-Enabled-Care” and their development within the iCARE-PD consortium, whose members had an interest in developing patient-centered solutions to improve the usability, acceptability, and future added value of each technology. Importantly, all 3 options had the potential to support and enhance PD self-management. This work does not intend to appraise all available digital solutions for PD. Readers are referred to these references [5-7] for a broader context on how these examples could integrate within the current, and expanding, ecosystem of digital solutions.

Despite differences among the 3 technologies evaluated, our objective was to assess their individual usability and acceptability using a unified approach, allowing us to implement a patient-centered strategy to enhance their development; it builds upon previous research [8]. While some overlap exists between the technologies, each was developed independently and has not yet been integrated into a common tool. Their primary purpose is to support self-care at different levels, though both their mutual integration and also the interplay with existing solutions remain to be determined.

Beyond evaluating usability and acceptability, another aim of this study was to critically examine the research process itself. By reflecting on our methodology, we seek to identify valuable lessons that can inform and improve future usability and acceptability studies for TEC development in the PD field and related areas.

This study fills an important gap in the literature by moving beyond individual assessments of digital health tools. While previous research has examined the feasibility and clinical benefits of TEC solutions, limited work has focused on a structured, patient-centered evaluation of multiple TEC solutions using a standardized methodology. By applying both quantitative usability metrics and qualitative user insights, this study offers a comparative perspective that highlights key usability challenges and best practices. Our findings provide a foundation for improving future TEC development and integration within a patient-centered PD care model.

## Methods

### Study Design

We conducted a multicenter international study in 5 tertiary PD centers: the Fundación Investigación HM Hospitales in Spain, the Ottawa Hospital Research Institute in Canada, the Charles University in the Czech Republic, the CNS-Campus Neurológico in Portugal, and the Mater Misericordiae University Hospital Dublin in Ireland between December 2020 and September 2023. The data from Ireland were not included in this study due to data protection issues.

In this study, we evaluated the usability and acceptability of 3 health technologies (MooVeo [9], PDMonitor [10], and SpiroGym [11]) with a focus on usability, user confidence, and user satisfaction.

### Description of the Technologies Included in the Study

MooVeo [9] is a software designed to assist physicians and people with PD by using a standard computer’s webcam. It has a technology readiness level of 6‐7 and it is intended to support disease monitoring in various settings: (1) response to treatment, (2) disease progression, or (3) diagnosis. The patient stands at a prespecified distance from a computer screen. MooVeo guides the patient through 3 simple motion tasks, detailing how to perform them through text and figures and recording videos of the different tasks. The software localizes different points on the hand and tracks them as the patient performs the task. The software generates various metrics, such as mean amplitude and speed of movement. A report is created with these data, which can be sent to either the patient or a clinician. The software can be run locally or as a cloud app in a secure HIPAA (Health Insurance Portability and Accountability Act)-/GDPR (General Data Protection Regulation)-compliant manner. MooVeo's clinical utility has not yet been completely proven, as it is still under development. MooVeo is designed to objectively evaluate PD motor signs using a standard webcam. It has been validated against Inertial Measurement Units, the movement disorder society (MDS)–Unified Parkinson Disease Rating Scale, and neurological diagnosis in prior work [9].

SpiroGym [11] is a software designed to help patients improve their motivation and adherence to a self-managed respiratory therapy program. It has a technology readiness level of 6‐7. SpiroGym is installed on a mobile phone or tablet. It requires the use of an external microphone that transforms the sound created by a respiratory therapy device (both the microphone and this device were included in the evaluation performed in this study) into a graph, to provide visual feedback to people with PD on the quality of their inspiratory or expiratory maneuvers. SpiroGym also creates training diaries and provides summary information, such as the mean strength and effective time of the maneuver, and long-term status of expiratory or inspiratory muscle and cough strength. For telemedicine purposes, the SpiroGym sends patients’ training results to their therapist via the internet. As mentioned, it could be used clinically both for an active respiratory therapy approach and for respiratory function tracking.

PDMonitor [10] is a Class IIa Medical Device (European regulation EE 93/42/EEC). The PDMonitor system (PD Neurotechnology Ltd) is a noninvasive, continuous monitoring system composed of a set of wearable monitoring devices (it has 5 different sensors), a patient mobile app, and a physician web and mobile app. PDMonitor devices are attached to the patient’s body in specific body positions (the shanks, wrists, and waist). This solution enables people with PD and their care partners to record data on medication, nutrition, and self-assessed motor and nonmotor status information (it includes basic questionnaires with free-text questions to keep track of these aspects as a secondary objective). The web app, called the “Physician Tool,” provides physicians reports on a number of motor and nonmotor PD features, based on the data collected by the wearable devices and the patient mobile app.

### Study Population

The study included individuals (1) diagnosed with PD according to the MDS criteria [12], (2) aged between 20 and 80 years, (3) who were willing to participate after providing informed consent, and (4) who were able to complete the necessary data collection instruments. Exclusion criteria ruled out (1) individuals unable to communicate independently, regardless of communication aids, or (2) those with significant non–PD-related comorbidities. The study was designed to detect 95% of possible user errors with an estimated probability of occurrence of 0.15% using a sample size of at least 30 participants [8]. For one technology, PD Monitor, due to the CE (Conformité Européenne)-mark requirements, only a limited number of certified translations were available. As a result, the sample size was smaller for this technology, as some of the participants' languages have not been included in the available certified manuals which are required for a CE-marked device.

### Experimental Set-Up

This study consisted of 3 phases over a 2-week period, including a screening/baseline visit, an intermediate phone call, and a final visit. During the screening/baseline visit, the patient received information about the study. After signing the consent, demographic and clinical data were collected, including time since diagnosis, Hoehn and Yahr stage, and MDS-Unified Parkinson Disease Rating Scale part III in the practically defined “ON” state. Study participants performed an initial on-site use of MooVeo. The other 2 technologies—PDMonitor and SpiroGym—were explained and then used according to the following timeline: week 1 - PDMonitor, week 2 – SpiroGym; MultimediaAppendices 1  2).

A phone call was made after Week 1 to inquire about any adverse events and to ensure data collection for MooVeo and PDMonitor. The second in-person visit occurred at the end of Week 2 with a similar schedule of assessments.

### Study Assessments

The System Usability Scale (SUS) [13] is a short, reliable tool for measuring usability. It consists of a 10-item questionnaire with 5 response options, from “Strongly agree” to “Strongly disagree.” The final score ranges from 0 to 100, corresponding to a percentile ranking. SUS scores above 68 are considered above average [14]. The iCARE-PD Questionnaire (Multimedia Appendix 3) was created by the iCARE-PD consortium, and it evaluated the user’s perspective on the use of each technology and has 2 parts. Part 1 evaluates the acceptability and usefulness of digital health technologies through 14 questions with 5 Likert-type response options ranging from “Strongly agree” to “Strongly disagree,” and a free-text response to provide additional information. Part 2 collected the user’s perspective on applying digital technology for self-care in daily life. Study participants chose one of 9 pairs of words that captured different attitudes toward each digital technology. Additionally, study participants could suggest design improvements for each digital health technology in a free-text format and comment on both positive and negative aspects. Data were collected via web using the REDCap (Research Electronic Data Capture; Vanderbilt University) database [15,16].

### Data Analysis

Descriptive statistical analyses were conducted. We did not compare the different technologies, so no inference techniques were used. The reason is that the 3 technologies are not comparable by nature: they offer dissimilar datasets, have different intended uses, and hold different regulatory stages. The analyses were performed using R (version 4.3.2; R Core Team) [17]. The qualitative data analysis was performed using thematic analysis techniques [18,19]. Two team members evaluated all raw responses in their entirety and performed independent iterations to identify the main themes. Subsequently, they identified subthemes and together, they agreed on the themes and subthemes they mutually identified before reanalyzing the answers and counting how many times they were mentioned.

### Data Management

Deidentified clinical data were collected and managed using REDCap software in compliance with data protection regulations applicable to study sites. A unique identification code was assigned to each participant.

### Ethical Considerations

The study was conducted according to the guidelines of the Declaration of Helsinki and approved by the Institutional Review Board (or Ethics Committee) of Instituto de Investigación HM Hospitales (protocol code 18.05.1245-GHM approved on 26 December 2018). Informed consent was obtained by each participant following Good Clinical Practice Guidelines (ICH E6 R2) and in accordance with the ethics committee's approval of the protocol. All participants provided written informed consent before enrollment in the study.

## Results

### Overview

A total of 43 people with PD were included in the study (MooVeo, n=33; SpiroGym, n=32; PDMonitor, n=16) from the participating countries (Canada: n=10, 23.3%; Czech Republic: n=10, 23.3%; Portugal: n=10, 23.3%; and Spain: n=13, 30.2%). Table 1 contains the demographic and clinical characteristics of study participants.

**Table 1.** Baseline demographic and clinical characteristics of study participants, divided by the different technologies.

Characteristic		Total (n=43)	MooVeo (n=33)	PDMonitor (n=16)	SpiroGym (n=32)
Age (years), median (IQR)		67.0 (59.9‐71.5)	67.7 (63‐71.5)	65.7 (58.7‐70.9)	68.1 (61.8‐71.5)
Female, n (%)		15 (35.7)	12 (37.5)	7 (43.8)	11 (35.5)
White ethnicity, n (%)		37 (88.1)	30 (93.8)	12 (80.0)	28 (90.3)
Time since PD^a^ diagnosis, years, median (IQR)		9.6 (5.0‐13.7)	7.5 (4.0‐11.0)	7.0 (4.9‐9.3)	7.0 (4.0‐11.3)
Hoehn and Yahr stage, n (%)					
Symptoms on one side only		4 (9.3)	2 (6.1)	1 (6.2)	3 (9.4)
Symptoms on both sides but no impairment of balance		29 (67.4)	23 (69.7)	12 (75.0)	24 (75.0)
Balance impairment. Mild to moderate disease		8 (18.6)	7 (21.2)	1 (6.2)	4 (12.5)
Severe disability, but able to walk or stand unassisted		2 (4.7)	1 (3.0)	2 (12.5)	1 (3.1)
MDS-UPDRS III^b^, median (IQR)		29.0 (19.0‐42.0)	29.0 (18.0‐42.0)	24.5 (18.3‐34.3)	24 (17.0‐37.0)

a PD: Parkinson disease.

b MDS-UPDRS III (the Movement Disorder Society-Sponsored Revision of the Unified Parkinson Disease Rating Scale, part III) scores in ON state.

### Usability

**Figure 1.**
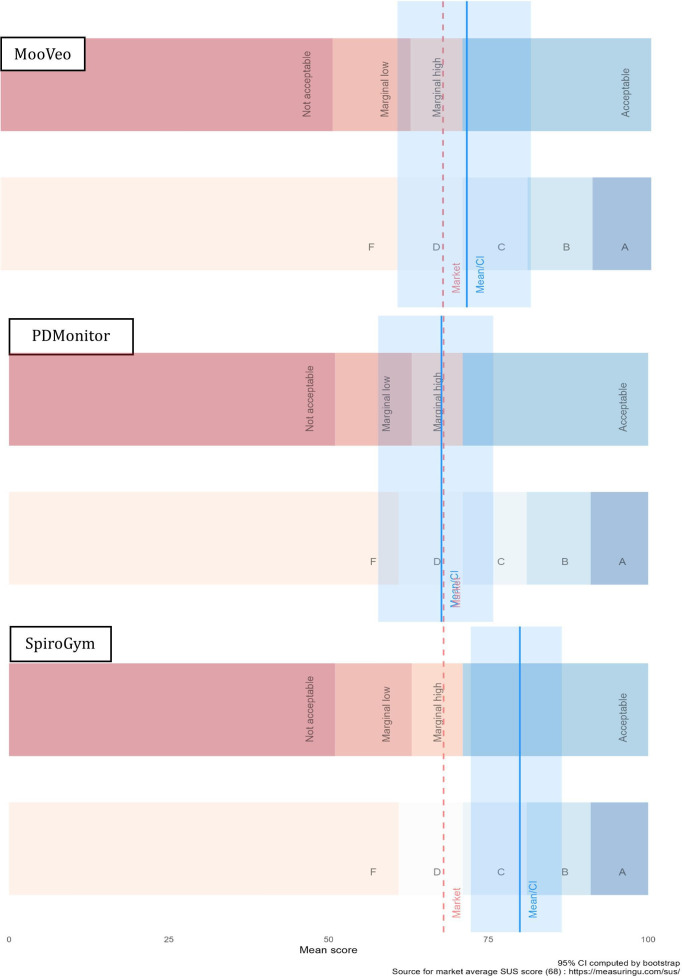
Graphical representation of the SUS mean score interpretation with market average comparison in the following order: MooVeo, PDMonitor, and SpiroGym. SUS: System Usability Scale.

The median SUS score was 75.0 (IQR 62.5‐82.5) for MooVeo, 73.8 (59.4‐78.1) for PD Monitor, and 82.5 (72.5‐87.5) for SpiroGym. After normalization using the grade proposed by Bangor et al [14], the average SUS score for each one was within the C (acceptable range; Figure 1).

**Figure 2.**
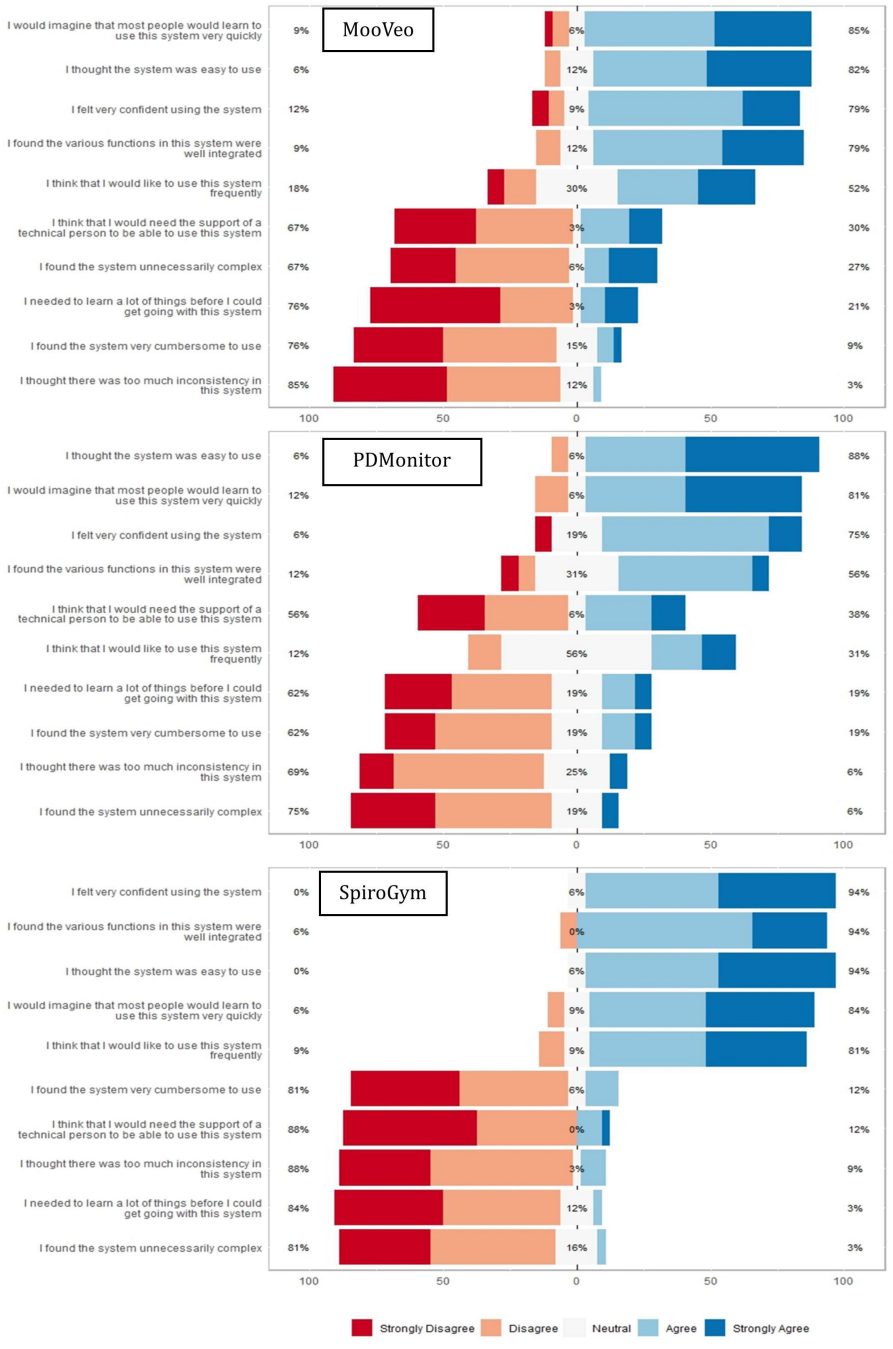
Likert graphs of System Usability Scale items for MooVeo, PDMonitor, and SpiroGym, showing the percentage of users who agreed (right of 0) and disagreed (left of 085) with each statement.

- MooVeo: A total of 28 (85%) users indicated that the system was easy to learn, 27 (82%) users agreed on its user-friendliness, and 26 (79%) users expressed confidence in its functionality and integration. Furthermore, 25 (76%) users did not perceive it as burdensome or requiring extensive learning prior to use, and 1 (3%) user found inconsistencies within the system (Figure 2).
- PDMonitor: A total of 14 (88%) users found the system easy to use, and 13 (81%) users believed it would be simple for others to grasp. Additionally, 12 (75%) users felt confident in its functionality, and 1 (6%) user felt the system was overly complex(Figure 2),
- SpiroGym: A total of 30 (94%) users reported finding the system intuitive to navigate, expressing confidence in its usability and seamless integration; 27 (84%) users believed others would quickly adapt to it; and 26 (81%) users indicated they would use it frequently. In contrast, only 3 (9%) users noticed inconsistencies, and 1 (3%) user perceived the system as overly complex or requiring extensive learning before use (Figure 2).

Individual responses to the SUS identified various areas of improvement (Figure 2), namely:

- MooVeo: A total of 6 (18%) users expressed a preference for sporadic use, and nearly 8 (25%) users found it complex. Additionally, 10 (30%) users indicated a need for technical assistance, and 7 (21%) users felt the need for a learning curve.
- PDMonitor: A total of 2 (12%) users reported a preference for sporadic use, and 6 (38%) users expressed a need for technical support. In addition, 2 (12%) users noted that the integration of functions could be improved, and 3 (19%) users felt they required extensive learning before using the system effectively.
- SpiroGym: A total of 4 (12%) users indicated a need for technical support and also 4 (12%) users found it cumbersome to use.

### Acceptability (iCARE-PD Questionnaire)

**Figure 3.**
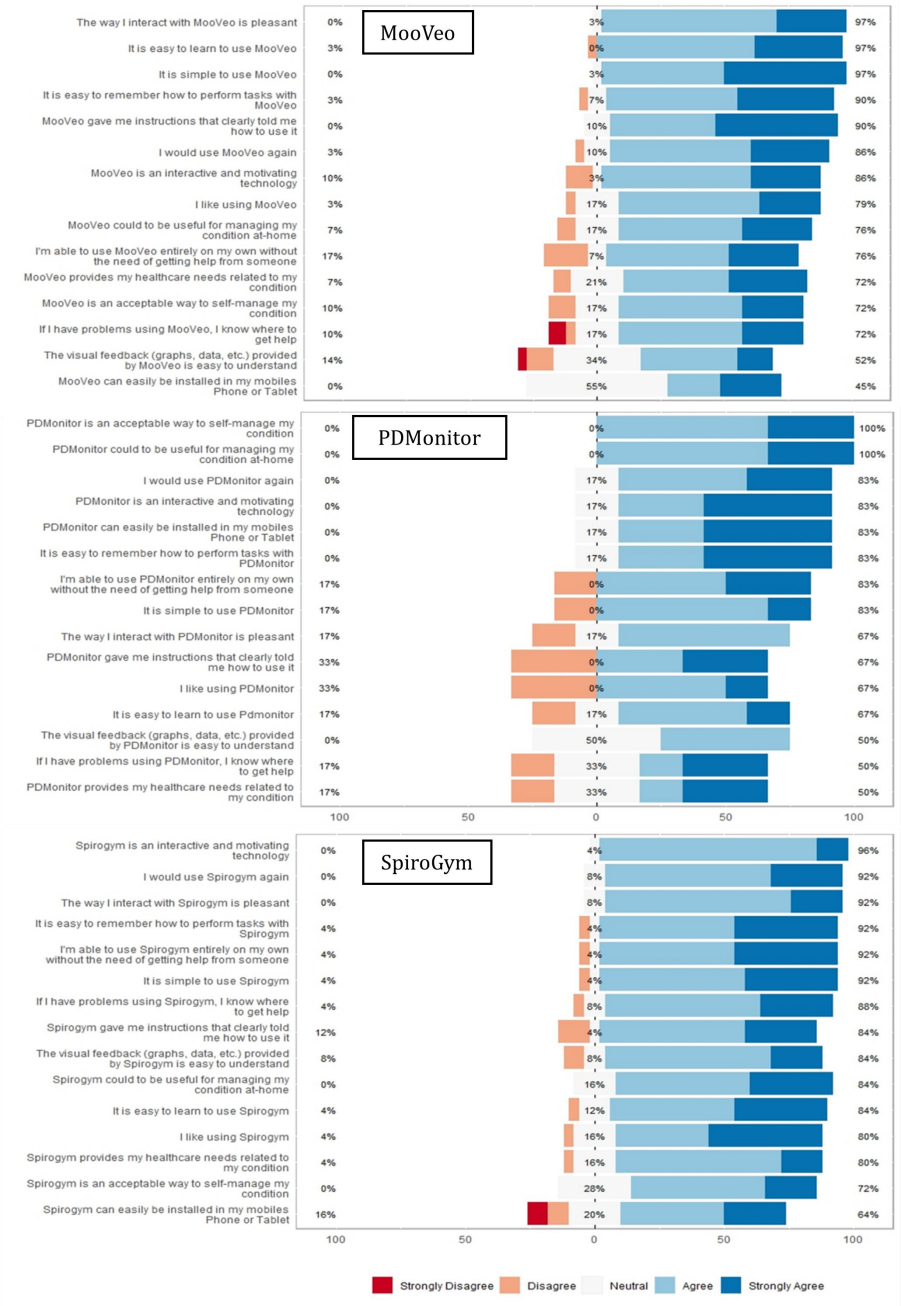
Likert graphs and iCARE questionnaire items for MooVeo, PDMonitor, and SpiroGym.

Study participants found the 3 technologies useful for managing their condition at home (MooVeo: n=25, 76%; PDMonitor: n=16, 100%; and SpiroGym: n=27, 84%) as well as interactive and motivating (n=28, 86%; n=13, 83%; and n=31, 96%; respectively). Additionally, they expressed willingness to use them again (n=28, 86%; n=13, 83%; and n=29, 92%; respectively), noting their ease of use (n=32, 97%; n=13, 83%; and n=29, 92%; respectively), ease of recall (n=30, 90%; n=13, 83%; and n=29, 92%; respectively), and independence in usage without external assistance (n=25, 76%; n=13, 83%; and n=29, 92%; respectively; Figure 3).

Areas of improvement as reported by study participants in the iCARE-PD survey included (Figure 3):

- MooVeo: A total of 24 (72%) users recognized its value in aiding self-management, and 15 (45%) users found the start-up process challenging. Further, 2 (7%) users felt that MooVeo fell short of their health care requirements, and 5 (14%) users suggested enhancements to the report for improved comprehension.
- PDMonitor: A total of 3 (17%) users expressed some initial concerns regarding its alignment with their health care needs. In addition, 3 (17%) users found the learning curve challenging and suggested improvements in user interaction for a more pleasant experience. Furthermore, while 8 (50%) users remained neutral about the graph visualization, 5 (33%) users expressed a desire for clearer instructions.
- SpiroGym: A total of 6 (18%) users expressed a desire for smoother installation processes and 4 (12%) users requested clearer instructions.

### Attitude Toward Technology Use

**Figure 4.**
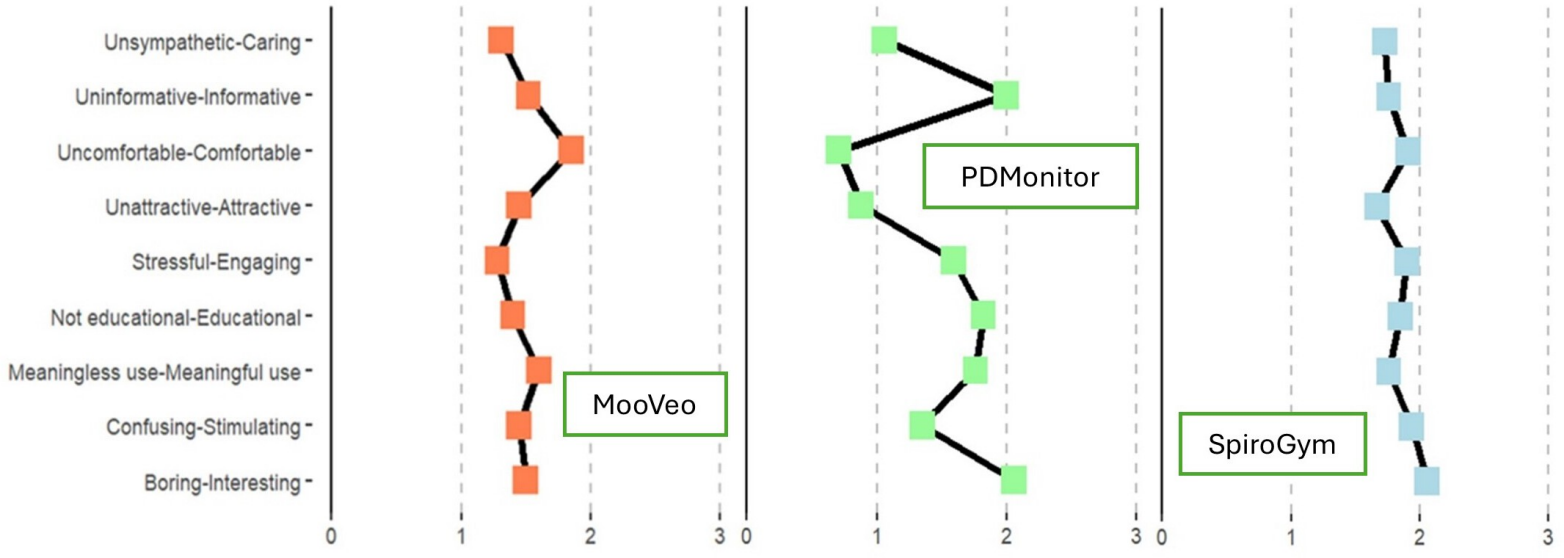
Pairs of attributes rated on a scale from -3 (left word) to +3 (right word), reflecting patient agreement. The graph starts at 0 (all results positive), with higher scores indicating stronger positive associations and greater acceptability. Technologies are color-coded: MooVeo (orange), PDMonitor (green), and SpiroGym (light blue).

The analyses of attitudes toward the use of each digital technology revealed that the 3 systems were generally perceived positively, with values ranging from 1 to 2 (scale range: −3, +3, Figure 4).

### Qualitative Analysis

Six themes were identified throughout the participants’ responses regarding positive and negative aspects of each technology and potential improvements: guidance (number of times identified=26), user interface (n=47), reports (n=22), usefulness (n=42), compatibility (n=24), and hardware (n=30). The 6 themes with the most relevant subthemes together with some examples can be seen in Table 2. The complete analyses can be found in Multimedia Appendix 4.

**Table 2.** The themes that emerged from the analysis are presented; the most relevant subthemes and some of the examples that support them are shown.

Theme and subtheme	Example user feedback
Guidance	
Clear instructions (n=24)	“There needs to be more information on the application. It can be confusing at first.” [MooVeo, user 2]
Intuitive (n=3)	“I like the feedback while training.” [SpiroGym, user 9]
Technical help (n=1)	“Create a chat where if a question arises, it is immediately solved.” [MooVeo, user 4]
User Interface	
User-friendly (n=45)	“This product is simple to use and has potential to be a useful tool.” [PDMonitor, user 6]
New functions (n=2)	“Simple and objective.” [MooVeo, user 19]
Reports	
Easy to understand (n=6)	“Easy to understand results.” [MooVeo, user 5]
Meaningful visualization (n=10)	“Improvements in the graph would be encouraging.” [SpiroGym, user 4]
Data quantity (n=6)	“Meaningful visualization.” [MooVeo, user 22]
Usefulness	
Informative (n=13)	“It provides feedback and confirms my impressions.” [PDMonitor, user 14]
Disease management (n=23)	“To have autonomy and independence to monitor myself.” [MooVeo, user 17]
Self-management (n=3)	“Self-management and autonomy for the realization of the exercises.” [SpiroGym, user 30]
Compatibility	
Platforms (n=3)	“The fact you can use it on your laptop or phone is easy.” [MooVeo, user 23]
Language/location (n=16)	“Part of the app was not translated.” [SpiroGym, User 25]
Functionality level (n=4)	“Devices do not interfere in your daily life.” [PDMonitor, user 5]
Hardware design	
Bands (n=18)	“Technology seems solid, equipment is comfortable to wear, wrist bands could be more flexible.” [PDMonitor, user 13]
Sensors (n=9)	“I would like smaller cables.” [SpiroGym, user 27]
Ergonomic (n=2)	“It was not possible for me to set up the mic on the machine as I can usually use it with one hand only. Needed help to put it on and remove it, and most patients will need help if they have motor symptoms. The actual device was then easy to use.” [SpiroGym, user 6]

## Discussion

### Principal Findings

In this study, we individually assessed the usability and acceptability of 3 health technologies developed within the iCARE-PD consortium. As stated in the introduction, a direct comparison among them was not intended, given their differing purposes and developmental stages. The SUS scores for all systems exceeded the threshold for market acceptance (SUS scores above 68 are considered above average or acceptable and a tech device is considered not acceptable if it goes below 50 points on the score [14]). This was further supported by item-level analyses indicating ease of learning, ease of use, and confidence in use. Additionally, areas for improvement were identified, particularly concerning user experience, technical support, and training materials. Users emphasized the need for simpler tasks, clearer instructions, and streamlined functionality, which should be taken into consideration. These findings highlight the importance of customer support in health care technologies, aligning with a 2022 study conducted in Germany on the PDMonitor technology [20,21].

Despite these challenges, users expressed a willingness to continue using the technologies, recognizing their value in independently managing their disease at home. The iCARE-PD survey revealed installation challenges, providing further insight into the technical difficulties previously mentioned and reinforcing the necessity of clear instructions for effective engagement.

Qualitative analysis identified key patient concerns, categorized into guidance issues, user interface, reports, usefulness, compatibility, and hardware. Notably, hardware aspects such as ergonomics were highly valued by patients and significantly influenced their evaluation of usability and acceptability. Overall, users prioritized ease of use, clarity of purpose, and robust technical support. While some patients did not clearly perceive the role of the technology in self-management, they acknowledged its potential utility within a broader disease management approach. Ensuring hardware adaptation to users’ needs and providing easily understandable results were also identified as critical factors for enhancing user experience and satisfaction with health care technologies.

There remains a scarcity of qualitative studies incorporating patients’ perspectives in technology development. Among the limited examples, our findings align closely with those of Grosjean et al [22], who conducted a qualitative analysis within our group, evaluating a “digital companion” for people living with PD to support personalized self-care. Their study identified 6 main issues: meaningful visualization, interactivity, usefulness, adaptability and personalization, compatibility, and guidance. The striking similarity of these findings to our results, despite differences in the technological solutions assessed, lends further credibility to our conclusions.

We propose this framework as a potential model for future technology studies that prioritize the patient’s perspective or, at the very least, incorporate this subanalysis into the overall development process. This approach could ensure that, by the time technological solutions reach clinical practice, they have already been tested and refined based on patient input.

As described in prior work for other wearable and similar technologies [23], for some participants, there were usability or acceptability issues identified when testing each solution. This work has allowed the development teams to gather unique insights that will help improve the usability experience, adherence, and utility for future users with PD, once these perspectives are integrated into each technology and validated through larger prospective efforts.

### Strengths and Weaknesses

A key strength of our study is its inclusion of patient perspectives in usability and acceptability assessments. The qualitative approach used here is rarely found in PD-related literature [24], and we believe that similar methodologies could benefit other technology development projects. We also included a variety of geographies and participants across the disease spectrum to make the study as globally representative as possible.

A significant challenge was the evaluation of 3 distinct TEC solutions under a unified approach. However, this reflects a more realistic scenario, as the implementation of multiple digital technologies appears more feasible than relying on a single one, bringing us closer to future real-world applications [7]. Although these digital technologies measure different aspects of PD, a broader range of them may be required to capture both motor and nonmotor critical aspects. Nonetheless, patient compliance and digital literacy remain significant concerns, particularly when multiple tech solutions must be worn over extended periods of time and their integration for PD management remains to be evaluated [7].

The use of these solutions did not always align precisely with their intended purpose. A notable example is PDMonitor, designed for continuous use over 3‐5 days per month, with its graphs intended for trained physicians rather than patients [10]. Consequently, patient perception of benefit in this study may have been influenced by the fact that PDMonitor effectiveness is inherently linked to timely, well-informed physician decisions. Previous research has demonstrated that continuous use and physician engagement with this technology can yield long-term benefits for patients [25].

A key limitation of this study is the relatively small sample size, which constrains statistical power and generalizability. Future research should aim to increase the sample of participants using these technologies. However, the cohort examined here provides a reasonable representation of a PD population with national diversity. Another limitation is the exclusion of individuals with PD who had significant comorbidities preventing them from using the studied technologies. As a result, caution should be exercised when generalizing findings to the broader PD population. Future projects should aim for greater inclusivity. Furthermore, while we did not stratify the analysis by disease stage in this study, future research could incorporate this approach to provide more nuanced insights. TEC solutions such as telemedicine and self-care applications could be particularly beneficial for patients with comorbidities who cannot visit the hospital, offering opportunities for regular televisits or teletraining.

To minimize ecological bias, technologies were used in participants’ homes. However, each technology was only used for 1 week. Future studies should evaluate long-term usability and acceptability, as an extended observation period may yield deeper insights into patient adherence and overall acceptability of these technologies. It remains unclear whether longer exposure would enhance usability and acceptability or reveal additional barriers. It remains unclear whether longer exposure would enhance usability and acceptability or reveal additional barriers. Moreover, other critical questions remain unanswered, including data on clinical utility [20] and perspectives from additional stakeholders such as health care providers, caregivers, and technical experts.

Finally, digital literacy was assumed in this study but was not explicitly evaluated. We acknowledge that this could represent a significant barrier to technology adoption, usability, and acceptability. To promote equitable access and effective implementation, future projects should include an evaluation of digital literacy across diverse patient populations.

### Conclusions

The enhancement of the methodology by which technology is introduced to patients will facilitate the integration of telehealth in clinical care, thereby improving disease management, increasing efficiency, providing enhanced access for all, and enabling the individualization of decision-making.

This article presents a comprehensive evaluation of the usability and acceptability of 3 technologies used in the treatment of patients with PD. The study illustrates how the strengths and weaknesses of the technologies were identified, including a qualitative analysis that revealed guidance, user interface, reports, usefulness, compatibility, and hardware as the primary concerns of the patients. These findings will inform the essential changes that are necessary to enhance the patients’ perception and use of our devices. We put forth this framework as a potential model for future technology studies that prioritize the patient perspective.

## Supplementary material

10.2196/67986 Multimedia Appendix 1 Chronology of the study and the data collected.

10.2196/67986 Multimedia Appendix 2 Chronology of the study and the data collected during each visit.

10.2196/67986 Multimedia Appendix 3 iCARE-PD Questionnaire example for Mooveo.

10.2196/67986 Multimedia Appendix 4 The number of times that each of the topics was identified in the 3 open-ended questions, broken down by technology and with the total number of times that they appear.

## Abbreviations

CE: Conformité Européenne
GDPR: General Data Protection Regulation
HIPAA: Health Insurance Portability and Accountability Act
MDS: movement disorder society
PD: Parkinson disease
REDCap: Research Electronic Data Capture
SUS: System Usability Scale
TEC: technology-enabled care
