# Supplementary figures and images for: Insights From a Mixed Methods Analysis of 3 Health Technologies Used in Patients With Parkinson Disease: Mixed Methods Study
Source: J Med Internet Res. 2025 Aug 1;27:e67986. doi: 10.2196/67986 (PMC12316440; doi:10.2196/67986)

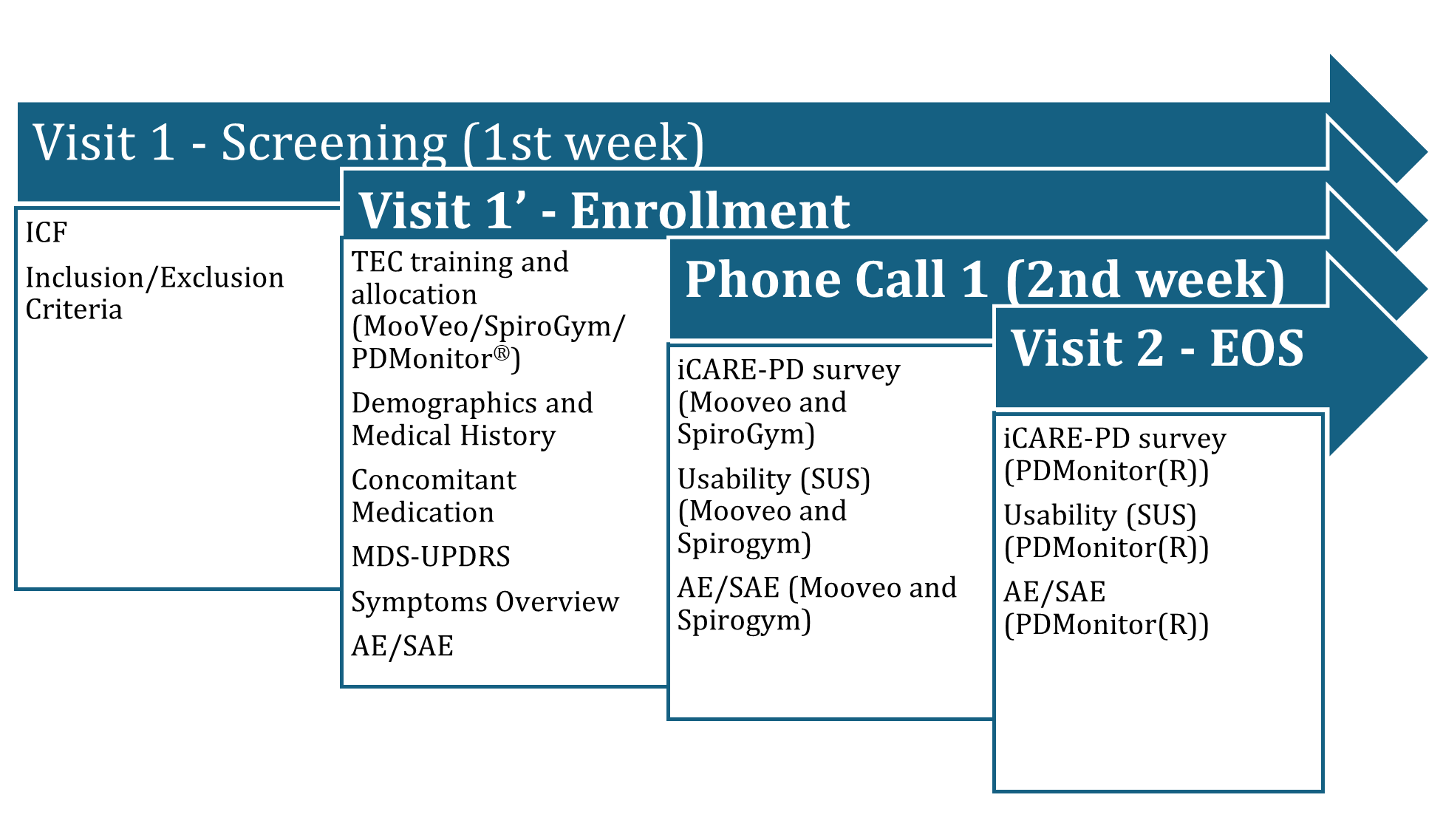

Supplement: Multimedia Appendix 1 [file jmir-v27-e67986-s001.png]
