# Supplementary material for: Insights From a Mixed Methods Analysis of 3 Health Technologies Used in Patients With Parkinson Disease: Mixed Methods Study
Source: J Med Internet Res. 2025 Aug 1;27:e67986. doi: 10.2196/67986 (PMC12316440; doi:10.2196/67986)
Supplement: Multimedia Appendix 2 [file jmir-v27-e67986-s002.docx]

| **Data Collection According to Standard Clinical Practice** | **Visit 1**  **Screening** | **Visit 1’**  **Enrollment** | **Phone Call 1** | **Visit 2**  **EOS** |
| --- | --- | --- | --- | --- |
| ICF | Χ |  |  |  |
| Inclusion/Exclusion Criteria | X |  |  |  |
| TEC training and allocation (MooVeo/SpiroGym/ PDMonitor^®^) |  | X |  |  |
| Demographics and Medical History |  | X |  |  |
| Concomitant Medication |  | Χ |  |  |
| MDS-UPDRS |  | X |  |  |
| iCARE-PD survey |  |  | X^a,b^ | X^c^ |
| Usability (SUS) |  |  | X^a,b^ | X^c^ |
| Symptoms Overview |  | X |  |  |
| AE/SAE |  | X | X | X |

**Table S1.** It displays the chronology of the study and the data collected during each visit. a) The study was conducted for MooVeo. b) The study was conducted for SpiroGym. c) The study was conducted for PDMonitor^®^.
