# Supplementary material for: Insights From a Mixed Methods Analysis of 3 Health Technologies Used in Patients With Parkinson Disease: Mixed Methods Study
Source: J Med Internet Res. 2025 Aug 1;27:e67986. doi: 10.2196/67986 (PMC12316440; doi:10.2196/67986)
Supplement: Multimedia Appendix 3 [file jmir-v27-e67986-s003.docx]

**Supplementary material**

Multimedia Appendix 3. iCARE-PD Questionnaire example for Mooveo

A. These questions are rated as follows: Strongly agree – Agree – Neither or N/A – Disagree – Strongly disagree

1. Mooveo provides my healthcare needs related to my condition.

2. It is simple to use Mooveo

3. It is easy to learn to use Mooveo

4. I like using Mooveo

5. If I have problems using Mooveo, I know where to get help.

6. Mooveo gave me instructions that clearly told me how to use it.

7. I'm able to use Mooveo entirely on my own without the need of getting help from someone

8. It is easy to remember how to perform tasks with Mooveo

9. The way I interact with Mooveo is pleasant.

10. Mooveo could to be useful for managing my condition at-home.

11. Mooveo can easily be installed in my mobiles Phone or Tablet.

12. The visual feedback (graphs, data, etc.) provided by Mooveo is easy to understand

13.Mooveo is an interactive and motivating technology.

14. Mooveo is an acceptable way to self-manage my condition.

15. I would use Mooveo again.

B. Listed below are several pairs of words. Place a check mark on the line between each pair to indicate what Mooveo means to you. Rated from -3 (left word) to +3 (right word):

1. Interesting-Boring

2. Meaningful use -Meaningless use

3. Informative-Uninformative

4. Engaging-Stressful

5. Comfortable-Uncomfortable

6. Stimulating-Confusing

7. Attractive-Unattractive

8. Educational-Not educational

9. Caring-Unsympathetic

C. Free-text questions:

1. This will be where you note positive feedback about the technology. What did you like?

2. This will be where you capture criticisms about the technology.

3. There you take down any ideas, suggestions to make improvements.
