# Supplementary material for: Insights From a Mixed Methods Analysis of 3 Health Technologies Used in Patients With Parkinson Disease: Mixed Methods Study
Source: J Med Internet Res. 2025 Aug 1;27:e67986. doi: 10.2196/67986 (PMC12316440; doi:10.2196/67986)
Supplement: Multimedia Appendix 4 [file jmir-v27-e67986-s004.docx]

|  | POSITIVE | | | | NEGATIVE | | | | IMPROVEMENTS | | | | **TOTAL** |
| --- | --- | --- | --- | --- | --- | --- | --- | --- | --- | --- | --- | --- | --- |
|  | M | P | S | TOTAL | M | P | S | TOTAL | M | P | S | TOTAL |  |
| **Guidance** | 3 | 0 | 4 | 7 | 6 | 1 | 4 | 11 | 3 | 1 | 4 | 8 | **26** |
| **User interface** | 13 | 6 | 13 | 32 | 3 | 0 | 2 | 5 | 4 | 3 | 3 | 10 | **47** |
| **Reports** | 2 | 0 | 1 | 3 | 4 | 0 | 6 | 10 | 5 | 1 | 3 | 9 | **22** |
| **Usefulness** | 12 | 8 | 10 | 30 | 2 | 1 | 3 | 6 | 4 | 1 | 1 | 6 | **42** |
| **Compatibility** | 1 | 2 | 3 | 6 | 5 | 2 | 6 | 13 | 2 | 2 | 1 | 5 | **24** |
| **Hardware** | 0 | 4 | 0 | 4 | 0 | 13 | 1 | 14 | 1 | 10 | 1 | 12 | **30** |

**Table S2.** The number of times that each of the topics was identified in the 3 open-ended questions, broken down by technology and with the total number of times that they appear.
